# 

**Published:** 1887-11-12

**Authors:** 


PRICE ONE PENNY.
No. 59, Vol. III.—November 12th, 1887.
[Registered at the General Post Office as a Newspaper.]

Per Annum, 6s. 6d., post free.
Monthly Parts, 6d.; post free, 7½d.
Ditto per Ann., 7s. 6d., post free.

### THE HOSPITAL

A WEEKLY INSTITUTIONAL JOURNAL OF

